# A Two-Year Participatory Intervention Project with Owners to Reduce Lameness and Limb Abnormalities in Working Horses in Jaipur, India

**DOI:** 10.1371/journal.pone.0124342

**Published:** 2015-04-21

**Authors:** Christine E. Reix, Amit K. Dikshit, Jo Hockenhull, Richard M. A. Parker, Anindo Banerjee, Charlotte C. Burn, Joy C. Pritchard, Helen R. Whay

**Affiliations:** 1 School of Veterinary Sciences, University of Bristol, Langford House, North Somerset, United Kingdom; 2 Help in Suffering, Maharani Farm, Durgapura, Jaipur, India; 3 Praxis Institute for Participatory Practices, C—75, South Extension, Part II, New Delhi, India; 4 Centre for Animal Welfare, Department of Clinical Veterinary Sciences, Royal Veterinary College, Hertfordshire, United Kingdom; 5 Animals in International Development, Banwell, North Somerset, United Kingdom; 6 The Brooke, London, United Kingdom; Auburn University, UNITED STATES

## Abstract

**Background:**

Participatory methods are increasingly used in international human development, but scientific evaluation of their efficacy versus a control group is rare. Working horses support families in impoverished communities. Lameness and limb abnormalities are highly prevalent in these animals and a cause for welfare concern. We aimed to stimulate and evaluate improvements in lameness and limb abnormalities in horses whose owners took part in a 2-year participatory intervention project to reduce lameness (PI) versus a control group (C) in Jaipur, India.

**Methodology/Principal Findings:**

In total, 439 owners of 862 horses participated in the study. PI group owners from 21 communities were encouraged to meet regularly to discuss management and work practices influencing lameness and poor welfare and to track their own progress in improving these. Lameness examinations (41 parameters) were conducted at the start of the study (Baseline), and after 1 year and 2 years. Results were compared with control horses from a further 21 communities outside the intervention. Of the 149 horses assessed on all three occasions, PI horses showed significantly (P<0.05) greater improvement than C horses in 20 parameters, most notably overall lameness score, measures of sole pain and range of movement on limb flexion. Control horses showed slight but significantly greater improvements in four parameters, including frog quality in fore and hindlimbs.

**Conclusions/Significance:**

This participatory intervention succeeded in improving lameness and some limb abnormalities in working horses, by encouraging changes in management and work practices which were feasible within owners’ socioeconomic and environmental constraints. Demonstration of the potentially sustainable improvements achieved here should encourage further development of participatory intervention approaches to benefit humans and animals in other contexts.

## Introduction

There are approximately 17.3 million horses in the 70 countries defined by the United Nations Food and Agricultural Organisation as ‘low-income food-deficit countries’ (LIFDCs) [1]. Most of these animals are used for draught or pack work to support the livelihoods of low-income owners [2,3]. Concerns about the welfare of these working equids has led to a number of non-governmental organisations (NGOs) acting within these countries to provide veterinary treatment, owner education programmes or other interventions with the ultimate aim of improving the welfare of working equids [3].

To successfully improve welfare in these populations, interventions must motivate the people who own or work with the horses to make changes to their own behaviour on behalf of the horse whilst not necessarily receiving any direct benefit from their actions themselves [4]. This can pose a greater challenge than interventions that work directly with the implementer who is also the direct beneficiary of any behaviour change such as in human health interventions. One method that has been adopted by a number of sectors, including human health and sanitation, agriculture, livestock and animal health involves stakeholders and community members working together to achieve a common-goal [5,6,7]. Such participatory methods have been used to investigate animal health and agricultural development issues since the 1980s, and the concept of people’s participation in investigating and solving their own issues is central to international development thinking and practice [8,9].

Compared with more unilateral educational or resource-provision approaches some potential advantages of a participatory intervention project which involves the owners of working horses in analysis of equine welfare issues include: a better understanding of local opportunities and constraints to welfare improvement, such as the living and working environment and socioeconomic context; active involvement of horse owners in identifying risk factors for poor welfare and locally feasible actions to reduce them; and increased peer pressure and support for equine welfare improvement activities within horse-owning communities. Despite such advantages, published evaluations of participatory interventions are rare [10,11], especially those relating to working equid health and welfare, although some field workers have been implementing them for many years [12,13,14,15,16].

One challenge to systematically evaluating the efficacy of such interventions is that standardisation of methods is only possible in the early stages of the project; thereafter, almost by definition, each community will find its own way of responding to initial stimulus. This inherent noise can mean that many independent replicates are required to detect effects, which can be logistically demanding. Moreover, control groups are necessary to verify whether improvements are really attributable to the intervention rather than to extraneous factors. This is especially true for interventions that are intended to be sustainable over long time periods, during which many factors unrelated to the study could affect results. Control groups are also necessary to rule out Hawthorne-like effects [17], where people’s knowledge that they are being monitored *per se* can affect their behaviour. By monitoring the control group as well as the intervention group, effects of monitoring can be separated from those of the intervention itself. Finally, the success of the intervention must be quantifiable in some way, which can be difficult in some contexts. The problems to be tackled here, equine lameness, is multifactorial but it can be measured both broadly and in detail, so lends itself as a potentially valuable example of participatory intervention research.

Studies assessing the welfare of working horses, mules and donkeys in Afghanistan, Egypt, Ethiopia, India, Pakistan and The Gambia have revealed a high prevalence of abnormal gait or lameness, ranging from 90 to 100% of animals assessed [2,18]. A study of 227 horses in India and Pakistan found that 98% had a gait abnormality in all four limbs and 87% had at least one limb scoring 3 or 4 on a lameness scale of 0–4 (sound—non-weight-bearing) [19]. Other clinical limb abnormalities with a high prevalence in that study population included chronic foot pathology (100% of animals), chronic joint disease (94% of animals) and digital flexor tendonitis in at least one limb (83% of animals). Locomotor pathologies are a major source of pain in horses and behavioural indicators of pain are widely recognised to include abnormal weight distribution and abnormal movement [20]; therefore it is reasonable to conclude that at any one time the welfare of a large proportion of the working horse population is compromised by limb-related pain.

The aim of this study was to investigate whether lameness and limb-associated abnormalities in working horses in Jaipur, India, could be reduced during implementation of a 2-year participatory intervention programme with their owners.

## Materials and Methods

This study was carried out in and around the city of Jaipur in north-west India between October 2007 and December 2009. It received ethical approval from the University of Bristol (Investigation number UB/07/026) and was compliant with Indian law regarding the ethical use of animals in science.

### Experimental protocol

Forty-two horse-owning communities took part in the study: 21 communities formed the participatory intervention (PI) group, identified by staff from the local supporting organisation, Help in Suffering (HIS). A control (C) group of 21 communities was selected by matching with PI communities for the type of work carried out by their horses and ensuring geographical proximity of PI and C communities to minimise any effects of locality on study findings. The study purpose and protocol were verbally explained to participants and informed consent to participate in the study was obtained verbally due to sensitivities regarding the literacy of some participants. The ethical committee gave their approval for this consent procedure. Once consent was obtained the participant was placed on the participant list which was kept confidential. Control groups were only briefed on the monitoring they would receive and not the intervention received by the other group.

A facilitator was selected from each PI community by its members together with project staff, according to key criteria identified in a planning meeting. The criteria included how well respected the facilitator was within their community, their expertise with equids and knowledge of welfare, and their ability to work impartially without any bias towards or against different groups within their community. Facilitators attended three training workshops (total 10 days), exploring a range of equine welfare and lameness-related issues using participatory rural appraisal (PRA) exercises. These were devised for the project in association with an Indian institute for participatory practices (PRAXIS) and based on methods and exercises in common use in the international development sector [9]. Exercises explored equine husbandry needs, such as feeding and working practices, and identified actions that horse owners could take to meet their animals’ needs and reduce risks for lameness and limb problems. Facilitators repeated these exercises and stimulated discussions in meetings with voluntarily participating horse owners in their own communities, held approximately every 1–2 months throughout the 2-year project and often co-facilitated by the project field coordinator (AD). The progress, or otherwise, of individual owners’ provision of equine welfare needs for their horses was recorded collectively by each group of owners on a monitoring chart, which remained with the community. The control group did not receive training, facilitation or visits as described above.

### Assessment of lameness and musculoskeletal abnormalities

A detailed lameness assessment was carried out on horses in both PI and C groups on three occasions: at the beginning of the study (‘Baseline’: October 2007 to February 2008); halfway through the study period (‘Mid-study’: October to December 2009); and at the end of the 2-year period (‘Final’: October to December 2009). Baseline assessments were conducted sequentially to ensure that horses in the PI group had their first assessment before any intervention had begun; consequently the majority of PI assessments were conducted earlier in the assessment period than those for the C horses. The remaining assessments (Mid-study and Final) were conducted randomly across the assessment period for both groups. The intention was to assess all horses in each community at each occasion, however due to a variety of factors including normal buying and selling of horses, deaths, horse aggression precluding examination, owner refusal to let their horse be examined and owner absences, this was not possible.

To maximise repeatability, all lameness examinations in all years were carried out by a single equine veterinary clinician (CER) with experience of examining working equids on the Indian subcontinent. The lameness examination used a protocol containing 11 descriptors followed by 41 parameters relating to gait, conformation, feet, limbs and spine (see Table 1) and took approximately 15 minutes per horse. This field examination was modified from a very detailed examination reported previously [19,21]. Three parameters (overall lameness score, thoracolumbar dorsi-/ ventroflexion and lateral lumbar flexion) were assessed at the ‘whole horse’ level; the remaining 38 were assessed separately for each limb. Most parameters were graded using subjective clinical judgement on a binary, 0–3, 0–4 or 0–5 scale. Owners of horses in need of treatment were referred to HIS for treatment. The clinician was not blinded to the study groups.

**Table 1:** Lameness examination protocol used for 862 horses working in Jaipur and its environs, India (adapted from [12]).

Parameter^1^	Scale	Description
*Lameness score*		
Limb lameness score	0–10	For each limb individually, lameness assessment at walk, based on head or hip movement, foot flight and placement, stride length and tracking up, joint flexion, toe drag. 0 = sounds; 10 = non-weight-bearing
Ataxia	0–1	0 = no ataxia, 1 = ataxic
Overall lameness score	0–10	Overall lameness assessment at walk using criteria as above. 0 = sound, 10 = unable to walk
*Limb conformation*		
Toe angle^2^	degrees	Toe-in (-ve value) or toe-out (+ve value)
Fetlock angle^2^	degrees	Fetlock varus (-ve value) or valgus (+ve value)
Hoof-pastern axis^2^	degrees	Broken backward (-ve value) or foreward (+ve value)
Carpus angle cranial view^2^	degrees	Carpal varus (-ve value) or valgus (+ve value)
Carpus angle lateral view^2^	degrees	Back at knee (-ve value) or over at knee (+ve value)
Sickle hock^2^	degrees	Lateral view
Cow hock^2^	degrees	Caudo-cranial view
Hocks touch or cross when standing square	0–1	Caudo-cranial view. 0 = hocks do not touch, 1 = hocks touch or cross
*Muscle atrophy or asymmetry*		
Triceps	0–3	0 = none, 1 = mild atrophy (only visible on close inspection), 2 = moderate atrophy (up to 50% of muscle mass), 3 = severe atrophy (50 to 100% of muscle mass)
Gluteal	0–3	As for triceps
*Hoof conformation*, *structure and quality*		
Mediolateral hoof balance	0–5	Overall assessment of mediolateral hoof balance: 0 = best^3^ hoof balance, 5 = worst^4^ hoof balance
Dorsopalmar hoof balance	0–5	Overall assessment of dorsopalmar hoof balance: 0 = best^3^ hoof balance, 5 = worst^4^ hoof balance
Heel collapse	0–3	Lateral view. 0 = no heel collapse, 1 = mild, 2 = moderate, 3 = severe
Dorsal hoof wall angle^5^	degrees	Measured at midline dorsal hoof wall
Hoof conformation		Overall assessment of hoof conformation, including length of toe, heels, hoof angle and shape: 0 = best^3^ conformation, 5 = worst^4^ conformation
Hoof wall quality upper half	0–5	Overall assessment of upper half of the external hoof wall, taking into account all aspects of the wall and any pathology present: 0 = best^3^ quality, 5 = worst^4^ quality
Hoof wall quality lower half	0–5	As for upper half
Sole structure	1–5	Assessment of overall degree of concavity or convexity of the sole of each foot: 1 = severely concave, 2 = mildly concave, 3 = flat sole, 4 = mildly convex, 5 = severely convex
Sole quality	0–5	Assessment of overall quality of sole, taking into account texture and any pathology present: 0 = best^3^ quality, 5 = worst^4^ quality
Frog quality	0–5	Assessment of overall quality of frog, taking into account texture and any pathology present: 0 = best^3^ quality, 5 = worst^4^ quality
Shoe fit	0–5	0 = no shoe present, 1 = best^3^ shoe fit, 5 = worst^4^ shoe fit (including incomplete shoes)
*Foot pathology*		
Pain on percussion^6^	0–3	Assessed separately at five points: craniomedial sole, craniolateral sole, centre of frog, medial heel, medial quarter of wall, lateral quarter of wall
Digital pulse	0–1	0 = normal, 1 = abnormal
*Limb palpation*		
DIP	0–3	Assessed for swelling and clinical assessment of chronicity.
		Swelling: 0 = no swelling, 1 = mild, 2 = moderate, 3 = severe
		Chronicity: 0 = no swelling, 1 = acute, 2 = sub-acute, 3 = chronic
MCP/MTP	0–3	As for DIP
DFTS	0–3	As for DIP
DDFT	0–3	As for DIP
SDFT	0–3	As for DIP
SL	0–3	As for DIP
Carpus/ tarsus	0–3	As for DIP
*Limb and spine manipulation*		
MCP/ MTP joint range of movement	0–4	0 = full range of motion possible, 1 = mild reduction in range of movement, 2 = moderate reduction, 3 = severe reduction, 4 = no joint flexion possible
Distal limb flexion pain response (MCP/MTP distal)	0–3	0 = no pain response, 1 = mild response, 2 = moderate response, 3 = severe response
Upper (FL)/ Full (HL) limb flexion range of movement	0–4	As for MCP/MTP joint
Upper (FL)/ Full (HL) limb flexion pain response	0–3	As for MCP/MTP joint
Thoracolumbar spine dorsoventral range of movement	0–4	As for MCP/MTP joint
Thoracolumbar spine pain on dorsiflexion/ ventroflexion	0–3	As for MCP/MTP joint
Lumbar spine lateral range of movement	0–4	As for MCP/MTP joint
Lumbar spine pain on lateral flexion (to left side)	0–3	As for MCP/MTP joint

^1^ Measured or assessed on all four limbs unless otherwise stated

^2^ Assessed with a visual measuring scale

^3^ Theoretical ideal

^4^ Based on all working horses examined to date by CER

^5^ Measured with hoof gauge

^6^ Assessed by response to digital pressure, hoof testers and percussion with a small hammer

DIP = distal interphalangeal joint; MCP = metacarpophalangeal joint; MTP = metatarsophalangeal joint; DFTS = digital flexor tendon sheath;

DDFT = deep digital flexor tendon; SDFT = superficial digital flexor tendon; SL = suspensory ligament; FL = forelimb; HL = hind limb

### Statistical analysis

The lameness assessment parameters were analysed as outcome variables in repeated measures multilevel statistical models [22] which controlled for the age group and type of work carried out by horses (added as fixed effects) and for the non-independence of data at the community-, horse- and (where applicable) limb-level, all added as random effects. The year (Baseline, Mid-study or Final), intervention group (PI or C) and their interaction were included as fixed effects.

Variables were transformed as appropriate to ensure the data better fitted the model’s assumptions and/or to facilitate model convergence (for example, combining ordinal categories with low prevalence). Ordinal response variables with a relatively large number of categories, such as the lameness score, were modelled as a continuous response variable if appropriate following transformation to Normal scores [23].

All statistical modelling was carried out using MLwiN v.2.22 [24]. Likelihood ratio tests and Wald tests were used to assess the significance of predictors; in keeping with similar studies [19], this is reported at a non-conservative level (p< 0.05) to avoid Type II errors (missing real effects).

## Results

### Sample sizes and data selection

In total, 439 horse owners participated in the study, 248 from the PI group of communities and 191 from the C group. Data were collected from all horses presented by each participating owner, although not all horses were assessed each year. Two PI communities (total 16 owners of 23 horses) did not wish to meet regularly as an equine welfare collective, so withdrew from the project during the first year, leaving 19 communities in the PI group and 21 in the C group. Excluding these, a total of 862 horses were assessed as part of the study on at least one occasion (PI group *n* = 448, C group *n* = 414). Analyses were conducted on data from horses assessed every year and remaining with the same owner throughout (*n* = 149; PI group = 83 animals belonging to 73 people in 18 communities, C group = 66 animals belonging to 58 people in 21 communities).

### Types of work carried out by horses in the study sample

Of the horses assessed on all three occasions, the majority (73% PI group, 80% C group) carried out ceremonial work (ridden by bridegrooms at weddings). The remaining horses transported goods by cart (14% PI group, 9% C group), transported people by cart (5% PI group, 2% C group), were foals (6% PI group, 8% C group) or carried out other types of work (non-ceremonial riding or unclassified) (2% PI group, 6% C group).

### Community responses to the intervention

The majority of community meetings occurred in the absence of members of the study team, but some themes were elucidated by observing a subset of meetings and via discussion with community members. Although intervention group discussions addressed general aspects of equine welfare, they focused on practices which could potentially reduce lameness and other limb abnormalities. These included changes in work practices, such as riding horses less frequently on rough tracks and riding more slowly, changes in use of local farriers and, where relevant, changes in cart maintenance (unpublished data).

### Lameness

All of the horses in both PI and C groups were scored as lame during at least one of the three annual lameness examinations. Overall, 4% of all lameness examinations indicated no lameness and of these, 79% were in horses less than 5 years of age. Horses from both groups demonstrated an improvement in average overall lameness scores between the Baseline and Final examinations, with the greatest change seen in the first year of the project (Table 2). This improvement was significantly greater in the PI group than the C group: PI horses improved by an average of 2 scores on a 0–10 scale (5.1 to 3.1), while C group horses started at a lower average score (4.3 out of 10) but improved by a mean of only 0.7, to 3.6 out of 10, at the Final examination. Changes in hind limb lameness scores reflected the same pattern as overall lameness scores, with the additional finding that PI horses improved from the Baseline to the Mid-study examination to a significantly greater extent than C horses. Forelimb lameness scores also improved over the 2 year period, although this did not differ significantly between PI and C groups.

**Table 2:** Significant differences in lameness and limb-related abnormalities between Baseline and Final examinations of 149 working horses belonging to 131 owners from Jaipur, India.

	Control (C) group summary statistics^2^				Participatory intervention (PI) group summary statistics^2^				Statistical validation	
Lameness/ limb parameter assessed^1^	Baseline assessment	Mid-study assessment	Final assessment	Magnitude and direction of difference between Baseline and Final^4^	Baseline assessment	Mid-study assessment	Final assessment	Magnitude and direction of difference between Baseline and Final^4^	Wald statistic	Significance of difference between PI and C groups (P value)
**Lameness**										
Overall lameness score (0–10)	4.3	3.4	3.6	0.7	5.1	2.8	3.1	**2**	11.07	<0.001
Limb lameness score—hindlimbs (0–10)	4.3	2.8	3.5	0.8	5.2	2.6	2.9	**2.3**	38.09	<0.001
**Forelimbs**										
*Limb conformation*										
Toe angle (° toe-in(-) to toe-out(+))	5.1	2.8	3.4	1.7	7.5	3.6	2.9	**4.6**	27.31	<0.001
Fetlock angle (° varus(-) to valgus(+))	1.6	0.5	1.3	0.3	6.2	0.5	1.2	**5**	142.84	<0.001
Hoof-pastern axis (° broken-back(-) to broken-forward(+))	-0.1	-0.5	0.1	-0.2	1.1	1	-0.3	**1.4**	5.49	0.019
Carpus angle cranial view (° varus(-) to valgus(+))	2.4	0.5	1	1.4	3.4	0.8	1.1	**2.3**	13.25	<0.001
*Hoof conformation*, *structure and quality*										
Hoof conformation (0–5)	3.7	3	3.1	**0.6**	3.4	2.9	3	0.4	5.1	0.024
Frog quality (0–5)	3.1	2	1.7	**1.4**	3.1	2.3	2.2	0.9	9.92	0.002
*Foot pathology*										
Pain response—any area of sole	42%	21%	36%	6%	61%	15%	23%	**38%**	19.03	<0.001
Pain response—craniomedial sole	27%	15%	22%	5%	38%	13%	15%	**23%**	7.51	0.006
Pain response—craniolateral sole	27%	12%	26%	1%	45%	10%	18%	**27%**	13.22	<0.001
Pain response—medial heel	19%	6%	11%	8%	35%	4%	9%	**25%**	4.14	0.042
*Limb palpation and manipulation*										
MCP joint range of movement (0–4)	1.6	1.5	1.6	**0**	1	1.1	1.3	-0.3	7.42	0.006
**Hindlimbs**										
*Limb conformation*										
Hoof-pastern axis (° broken-back(-) to broken-forward(+))	4.8	2.8	3.8	1	6.6	3.5	3.5	**3.1**	4.28	0.039
*Muscle atrophy or asymmetry*										
Gluteal	40%	10%	24%	16%	46%	9%	14%	**32%**	4.39	0.036
*Hoof conformation*, *structure and quality*										
Sole structure (1 (concave) to 5 (convex))	2	2.2	2	**0**	1.9	1.9	2.2	-0.3	15.26	<0.001
Frog quality (0–5)	3.1	1.9	1.3	**1.8**	2.9	2.2	1.7	1.2	9.54	0.002
Shod^5^	29%	19%	19%	**10%**	32%	20%	38%	-5%	5.79	0.016
*Foot pathology*										
Pain response—any area of sole	32%	7%	18%	14%	59%	12%	19%	**40%**	8.92	0.003
Pain craniolateral sole	15%	2%	10%	5%	40%	7%	8%	**32%**	9.13	0.003
Pain centre of frog	10%	2%	6%	4%	24%	5%	4%	**21%**	4.94	0.026
*Limb palpation and manipulation*										
MTP swelling (0–3)	2.1	2	2	0.1	2.2	2.1	1.9	**0.3**	6.43	0.011
Tarsal joint swelling (0–3)	2	1.5	1.7	0.3	2	1.6	1.6	**0.4**	6.85	0.009
Full limb flexion range of movement (0–3)	2	1.4	1.4	0.6	2.5	0.8	1.2	**1.3**	29.61	<0.001
Full limb flexion pain response	1.1	0.9	0.9	0.2	1.8	1.5	0.6	**1.2**	57.6	<0.001
**Spine manipulation**										
Lumbar spine pain on lateral flexion (to left side)	53%	18%	40%	13%	69%	16%	33%	**36%**	4.95	0.026

(Participatory intervention group n = 83 horses belonging to 73 people; Control group n = 66 horses belonging to 58 people). The group with the largest relative improvement between Baseline and Final has the text depicting the magnitude of change in bold.

^1^ See Table 1 for scoring methods

^2^ For continuous or ordinal data = mean of horses (for whole horse measures) or limbs (for limb-specific measures) affected. For binary measures or those aggregated into two groups for analysis = percentage of horses/ limbs affected

^3^ Relative improvement: the group which showed the most positive clinical change and/or the least negative clinical change (except for 'Shod' and 'Active response when approached' where no clinical value was applied)

^4^ Calculated as Year 1—Year 3, i.e. a positive value = clinical improvement; a negative value = deterioration (except for 'Shod' and 'Active response when approached' where no clinical value was applied)

^5^ 1st-order MQL estimation (otherwise, less-biased 2nd-order PQL estimation was employed in all binary and multinomial response models)

Overall lameness scores increased with age in both groups. Work type influenced lameness, with the highest scores seen in horses transporting people by cart, followed by those transporting goods by cart, followed by ceremonial/riding/other uses, and foals had the lowest lameness scores. Body condition score (BCS) was significant as a quadratic function, revealing a U-shaped relationship with overall lameness scores: a BCS of 3 (medium) was associated with the lowest overall lameness scores, whilst a lower (thinner) or higher (fatter) BCS was associated with higher overall lameness scores, highest when BCS was low.

### Hoof conformation, structure, quality and pain responses

The prevalence of pain responses to light percussion of the sole reduced to a considerably greater extent in PI horses than C horses (Table 2). Conversely, C group horses showed a greater improvement in frog quality and to a lesser extent forelimb hoof conformation than intervention horses. Whilst hoof wall quality and shoe fit did not differ significantly between groups, the proportion of shod hind feet increased from 32 to 38% in the PI group between the Baseline and Final examinations (with a fall to 20% at the Mid-study examination), and fell for the C group from 29% to 19% over the same period.

### Limb conformation

The mean forelimb toe angle remained positive throughout (toed out), although it decreased significantly in both groups across the 2-year period. The extent of this change was slightly greater for PI horses, which had a more toed-out conformation than C horses at the Baseline examination and were less toed-out than C horses at the Final examination. There was a greater degree of fetlock valgus in PI horses than in C horses at the Baseline, with valgus reduced significantly more for PI horses than C horses (mean 5° vs 0.3°) across the 2-year period, becoming comparable to that of C horses in the later assessments.

### Muscle atrophy

The prevalence of some degree of gluteal atrophy or asymmetry (mild to severe) reduced in both groups across the 2-year period, with a significantly greater reduction for horses in the PI group than for horses in the C group (31.7% vs 16.4%).

### Limb swelling, pain and range of joint movement

Hind limbs showed the greatest number of significant differences between intervention and control groups of horses in the extent of change in limb swelling, pain and range of joint movement. The average improvement in tarsal and metatarsophalangeal swelling between Baseline and Final examinations was higher in the PI than the C group, although relatively small in both groups. PI horses also demonstrated significantly more improvement in range of movement and reduced pain on joint flexion than C horses; in most cases the greatest improvement occurred within the first year of the study.

### Symmetry of lameness and clinical limb abnormalities

The pattern of uni- and bilaterality of fore- and hind limb lameness differed significantly between PI and C horses over time. For horses in the PI group, there was greater disparity in lameness scores between left and right limbs at the Baseline examination than for horses in the C group, although the opposite was true in the Final examination.

In the PI group, forelimb deep digital flexor tendon swelling and a reduced range of upper limb joint flexion were more likely to be unilateral at the Final examination than the Mid-study examination, whilst for the C group they were more likely to be bilateral at the Final examination than the Mid-study examination). No significant differences between the two groups were seen in the first year of the study.

## Discussion

This investigation aimed to identify whether 2-year participatory intervention project with working horse owners would lead to changes in lameness and limb abnormalities in their horses. The majority of horses in the sample population were used for ceremonial work which may be perceived as less taxing than other work types undertaken by working equids. However, the study period (October-December/February) each year coincided with the wedding season in Jaipur, which is the busiest time of year for ceremonial horses. The horses are ‘fattened up’ quickly prior to this period (often with large amounts of concentrates/oil leading to the painful foot condition laminitis), and then ridden very quickly on roads to weddings all over the state, during this busy time, resulting into acute tendon injuries and other limb problems.

The prevalence of lameness and clinical limb abnormalities in this study population was high, as found in previous studies of working horses [2,21], reflecting ongoing pain and contributing to poor welfare over prolonged periods of time. A significantly greater improvement in overall lameness score in the PI group of horses compared to the C group suggests a potentially positive effect of the intervention on reducing lameness. This finding was reflected in the hind limb (but not the forelimb) individual limb lameness scores, which may indicate that forelimb lameness is harder for the owners of working horses to influence, particularly in this sample population where most horses were carrying out ridden rather than driving work. Moreover, it was reflected in significant improvements in 19 other parameters compared with only two parameters that worsened slightly in the PI horses and just four parameters that improved in the C horses (Table 2).

In a different, larger sample of working horses, mules and donkeys (*n* = 10,843 across nine countries in the Global South), the prevalence of sole surface abnormalities and swollen tendons or joints followed similar patterns to the prevalence of gait abnormalities, which was unsurprising as they would be expected to contribute to lameness [18]. In the current study, sole pain and some measures of joint swelling and range of joint movement (such as metatarsophalangeal swelling, hind limb stiffness and pain responses to flexion of the limbs) followed similar patterns to the changes in lameness, although mean changes in some of these indices were relatively small. In contrast, there were no significant differences between PI and C horses in other measures of sole abnormality, except frog quality (fore and hind) and hind sole structure, both of which showed a greater improvement in the control than the intervention group. It is possible that the latter measures did not necessarily reflect pain or significant loss of function, while an accumulation of small improvements in several clinical conditions associated with pain led to the observed positive effect on overall and hind limb lameness scores in the intervention group.

The observed differences in limb conformation between PI and C horses over the 2-year project are unlikely to reflect changes in conformation *per se*, but to demonstrate changes in stance as a result of modified management practices such as hoof trimming and shoeing. PI horses showed a significantly greater change in hoof-pastern axis towards normality, although both the overall change and between-groups effect in the forelimbs were small. In the forelimbs, the toe angle improved significantly more (from toe-out towards straight) in the PI than the C horses, despite starting from a more abnormal baseline. Both of these, combined with the finding that significantly more PI than C horses had hind shoes at the Final examination compared to Baseline measures, indicate an increased uptake of farriery services in the intervention group compared with controls, so could reflect improved foot trimming. However, farriery practices are highly variable in this region and some can lead to increased rather than decreased limb and foot pathology [19] so, as expected, the observed changes in both groups demonstrate a complex relationship between patterns of shoeing and the changes in sole structure and quality described above.

This study found a U-shaped relationship between body condition score (BCS) and lameness, with the lowest lameness scores seen at BCS 3 (on a scale of 1–5) and higher lameness scores associated with both higher and lower BCS. This is worthy of further research as it differs from other studies which only found higher lameness scores in thinner horses [2,19]. The relatively high number of ceremonial ridden horses may explain the increase in lameness with high BCS: these animals are often kept in good to fat condition as previously noted and are at risk from laminitis [25].

This paper presents statistical differences between intervention and control groups of horses over the whole 2-year period; in this exploratory study the biological significance of small changes is unclear. Interesting patterns appear when comparing changes over the first year of the project (Mid-study vs Baseline examinations) with the second year (Final vs Mid-study examinations). Most showed significant improvement over the lifetime of the project and often the greatest observed change occurred in the first year, and then remained fairly constant or even deteriorated slightly in the second year. While the significant additional improvement in the PI compared to the C horses in the first year could be attributed to the effect of the participatory intervention, there could be several explanations for this pattern of change. Factors external to the project may have varied across years, such as changes in road surfaces or the seasonal availability of work, enabling improvement in lameness in the first year followed by inability to continue improving to the same extent. Alternatively this could reflect internal, project-related factors such as a higher engagement with the intervention process by horse owners and/or the project facilitator in the first year, followed by either habituation and less effort towards change in the second year, or a plateau effect in improvement once the easier management practice changes had been made, leaving only the more challenging risk factors to influence [26]. For example, average overall lameness score of horses in the PI group fell from a Baseline score of 5.1 out of 10 to 2.8 out of 10 Mid-study, followed by a slight increase to 3.1 out of 10 at the Final examination. This could reflect an intervention effect reducing the prevalence of acute, reversible limb and foot conditions in the early stages of the project, leaving the more chronic or irreversible conditions contributing to residual lameness, which would not be expected to improve to a great extent over the following year. A third possibility is that the increase in prevalence of shod hind feet at the Final examination could be associated with a plateau in improvement in lameness scores, by introducing farriery-related problems to previously unshod limbs.

A limitation to this study is that drift in either measurement technique or scoring may occur when observations are made at one-year intervals, although a set of guidance notes was used to reduce this risk. Intra-observer ratings of equine lameness can be reliable when repeated at 3-monthly intervals over a 9-month period [27] but longer intervals have not been tested. Furthermore, it was not logistically feasible for the observers to be blind to intervention and control groups, although this would clearly have been optimal.

Changes in lameness and limb abnormalities occurred in both PI and C horses and the patterns of change between the first and second years of the project were seen in control as well as intervention groups, although often to a lesser extent. This suggests that, as would be expected, external environmental or socio-cultural factors were influencing findings across the whole project area. There may also be internal, project-related reasons for similarities between PI and C groups. The mere presence of an assessor in control communities once a year, focusing on limbs and lameness, may have influenced C group owners to take some action, a phenomenon known as the Hawthorne effect [28]. Also, when designing a study involving regular communication and discussion with and between horse owners, geographic proximity of intervention and control groups leads to a trade-off between the desire to reduce variability in external, environmental influences (lower when intervention and control groups are geographically close) and the natural spread of intervention information between intervention and control groups (higher when the groups live or work in relatively close proximity). When selecting PI and C communities, these trade-offs were recognised and combined with other considerations such as matching by equine work-type. Although some changes seen in C horses may be due to ‘leakage’ of the participatory intervention or the effect of annual assessments alone, in terms of the overall aim of improving equine welfare this is a positive outcome in applied settings: farmer-to-farmer participatory extension of animal health and welfare messages and practices is a recognised tool within behaviour change projects [8].

This study appears to be the first of its kind to quantify the impact of a participatory intervention on an animal welfare issue. It demonstrates that field research in ‘real life’ situations, with dynamic groups of animal and human participants, is both valuable and feasible. In the context of evaluating human health promotion activities “the more powerful forms of health promotion action are those which are long term, and least easily predicted, controlled and measured by conventional means” [29]. Research and evaluation of health promotion pass through a series of stages in which outcomes should be understood first, followed by an increasing focus on the processes which led to those outcomes, in order to be able to repeat and refine projects and reproduce successes in other situations. This study fulfils the first step of this process by improving our understanding of lameness in working horses.
